# Evaluation of Image Quality for High Heart Rates for Coronary Computed Tomographic Angiography with Advancement in CT Technology: The CONVERGE Registry

**DOI:** 10.3390/jcdd10090404

**Published:** 2023-09-19

**Authors:** Ayman Abdelkarim, Sion K. Roy, April Kinninger, Azadeh Salek, Olivia Baranski, Daniele Andreini, Gianluca Pontone, Edoardo Conte, Rachael O’Rourke, Christian Hamilton-Craig, Matthew J. Budoff

**Affiliations:** 1 Department of Medicine, Lundquist Institute, Torrance, CA 90502, USA; aabdelkarim@dhs.lacounty.gov (A.A.); olivia.baranski@lundquist.org (O.B.); 2 Centro Cardiologico Monzino, IRCCS, 20138 Milan, Italygianluca.pontone@cardiologicomonzino.it (G.P.);; 3 Department of Clinical Sciences and Community Health, Cardiovascular Section, University of Milan, 20126 Milan, Italy; 4 Department of Medical Imaging, The Prince Charles Hospital, Brisbane, 4032 QLD, Australiachamiltoncraig@gmail.com (C.H.-C.)

**Keywords:** coronary artery disease, computed tomography, motion artifact, tachycardia

## Abstract

Objective: This study aims to evaluate image quality in patients with heart rates above or equal to 70 beats per minute (bpm), performed on a 16 cm scanner (256-slice General Electric Revolution) in comparison to a CT scanner with only 4 cm of coverage (64 slice Volume CT). Background: Recent advancements in image acquisition, such as whole-heart coverage in a single rotation and post-processing methods in coronary computed tomographic angiography (CCTA), include motion-correction algorithms, such as SnapShot Freeze (SSF), which improve temporal resolution and allow for the assessment of coronary artery disease (CAD) with lower motion scores and better image qualities. Studies from the comprehensive evaluation of high temporal- and spatial-resolution cardiac CT using a wide coverage system (CONVERGE) registry (a multicenter registry at four centers) have shown the 16 cm CT scanner having a better image quality in comparison to the 4 cm scanner. However, these studies failed to include patients with undesirable or high heart rates due to well-documented poor image acquisition on prior generations of CCTA scanners. Methods: A prospective, observational, multicenter cohort study comparing image quality, quantitively and qualitatively, on scans performed on a 16 cm CCTA in comparison to a cohort of images captured on a 4 cm CCTA at four centers. Participants were recruited based on broad inclusion criteria, and each patient in the 16 cm CCTA arm of the study received a CCTA scan using a 256-slice, whole-heart, single-beat scanner. These patients were then matched by age, gender, and heart rate to patients who underwent CCTA scans on a 4 cm CT scanner. Image quality was graded based on the signal-to-noise ratio, contrast-to-noise ratio, and on a Likert scale of 0–4: 0, very poor—4, excellent. Results: 104 patients were evaluated for this study. The mean heart rate was 75 ± 7 in the 4 cm scanner and 75 ± 7 in the 16 cm one (*p* = 0.426). The signal-to-noise and contrast-to-noise ratios were higher in the 16 cm scanner (*p* = 0.0001). In addition, more scans were evaluated as having an excellent quality on the 16 cm scanner than on the 4 cm scanner (*p* < 0.0001) based on a 4-point Likert scale. Conclusions: The 16 cm scanner has a superior image quality for fast heart rates compared to the 4 cm scanner. This study shows that there is a significantly higher frequency of excellent and good studies showing better contrast-to-noise and signal-to-noise ratios with the 16 cm scanner compared to the 4 cm scanner.

## 1. Introduction

Recent advancements in hardware and software have improved the imaging quality of coronary computed tomographic angiography (CCTA). The CONVERGE registry has demonstrated improved contrast uniformity, reduced noise, misalignments, and motion artifacts when using the Revolution 16 cm CT scanner compared to prior generations of CCTA, such as the 4 cm scanner, VCT (General Electric, Milwaukee, WI, USA) [1,2]. These improvements are due in part to faster gantry rotation speeds, increased temporal resolution, high definition detectors, ‘whole heart’ scanning, iterative reconstruction, and software-based motion-correction methods. Patients with high heart rates have historically been excluded from these analyses due to concerns of poor image acquisition complicated by motion and misalignment artifacts. However, with advancements in CT technology, there is a growing need to evaluate the performance of the 16 cm scanner in patients with heart rates above or equal to 70 beats per minute (bpm) in comparison to the 4 cm scanner. Therefore, the purpose of this study is to evaluate image quality in patients with heart rates above or equal to 70 beats per minute (bpm) in the 16 cm scanner in comparison to the 4 cm scanner.

## 2. Methods

### 2.1. Patient Population

This study prospectively consented and evaluated patients who underwent coronary CTA studies with a 16 cm scanner (Revolution CT, GE Healthcare, Milwaukee, WI, USA) at multiple sites as part of the international, multicenter CONVERGE Registry in accordance with the IRB-approved protocol. The intent of the CONVERGE research program is to develop an international registry database with Revolution CT users and to utilize this database to evaluate and quantify the extent to which the latest advancements in CT technology and post-processing applications address the challenges faced with cardiac CT. Sites included Lundquist Research Institute at Harbor UCLA Medical Center in Torrance, CA, USA; Centro Cardiologico Monzino, IRCCS, Milan, Italy; The Prince Charles Hospital, Brisbane, Queensland Australia; and Baptist Hospital of Miami Florida. We compared consecutive patients who underwent CCTA with heart rates above or equal to 70 bpm using a 16 cm scanner with a cohort of images captured on a 4 cm scanner (VCT, GE Healthcare, Milwaukee, WI, USA) from age-, gender-, body-mass-index, kilovoltage- and heartrate-matched subjects. Scans were evaluated at our site, Lundquist Research Institute at Harbor UCLA Medical Center in Torrance, CA. Consecutive patients were screened, enrolled, and consented to the CONVERGE Registry study, in accordance with the IRB-approved protocol. A total of 52 patients underwent CCTA using the 16 cm CT scanner versus 52 patients who underwent CCTA using the 4 cm CT scanner.

Patients with a left ventricular ejection fraction <40%, chronic kidney disease (estimated glomerular filtration rate <60 mL/min/1.73 m^2^ within 30 days of the CT), intravenous contrast allergy, or underlying atrial fibrillation were excluded from this study.

### 2.2. Patient Preparation

An oral and/or intravenous beta blocker or non-dihydropyridine calcium channel was administered if the patient presented with a heart rate above 70 beats per minute to achieve a heart rate under 70 beats per minute; however, in this subset of selected patients, further therapy was limited if it was deemed no longer clinically safe to do so, based on symptoms such as lightheadedness or dyspnea, or concerns of hypotension.

### 2.3. Imaging Acquisition

GE VCT 4 cm scanner: 64 detectors, 100 or 120 kVp, 430 mA, 350 ms/per rotation with 227 ms in temporal resolution, with 0.625 mm slice thicknesses. Electrocardiographic triggering was employed, so that each image was obtained at the same point in diastole, corresponding to 75% of the RR interval. The tube current ranged between 122 and 740 mA based on the patient’s BMI.

GE Revolution 16 cm 256 slice scanner: 256 detectors, 100 or 120 kVp, 400 mA, 280 ms/rotation with 140 ms temporal resolution, with 0.625 mm slice thicknesses. The tube current ranged between 122 and 740 mA based on the patient’s BMI. A medium field of view was selected for all patients. The scanner is equipped with an “autogating” capability, which automatically adjusts HR-dependent settings for triggered acquisition and gated reconstruction. Autogating was used to automatically acquire diastolic phases for lower HRs and both systolic and diastolic phases for higher heart rates. Electrocardiographic dose modulation, which reduces mA for nontarget phases, was used in these high HR acquisitions. All acquisitions were prospectively gated. Each scan was conducted in a single-beat acquisition within 1 cardiac cycle. No table movement was required due to the wide volumetric acquisition. Motion correction software (SnapShot Freeze; GE Healthcare) was used for correcting motion artifacts.

Following a scout radiograph of the chest (anteroposterior and lateral), a timing bolus using 10 to 20 mL of iodinated contrast (Visipaque, GE Healthcare, Buckinghamshire, UK) was performed to detect the time to optimal contrast opacification in the axial image at a level immediately superior to the ostium of the left main artery. Nitroglycerine 0.4 mg was sublingually administered immediately before contrast injection, unless contraindicated.

The contrast injection was performed using a power injector (Stellant; Medrad, Warrendale, PA, USA) through an antecubital vein at a rate of 5.0 mL/s. A triple bolus injection protocol was utilized. Patients were given an injection of 60 cc of contrast in the first phase, followed by 20 cc of contrast and 30 cc of saline in the second phase, completed by 50 cc of saline. All images were anonymized, transferred to a Core Laboratory for evaluation on a single vendor workstation to provide standardized postprocessing for both scanner acquisitions (AW 4.7, GE Healthcare). An SCCT level-3 trained CT cardiologist read all CT scans at a central reading center (Lundquist Research Institute at Harbor-UCLA in Torrance, CA, USA).

### 2.4. Image Reconstruction

For both scanner types, data reconstruction was performed using 0.625-mm-thin reconstructions with intervals ranging from 60% to 80%, and most of the data was reconstructed at 75% of the R-R phase. Adaptive statistical iterative reconstruction (ASIR-V) was used for 84 subjects or 80% of cases. The use of ASIR-V, including percent levels, is detailed in Table 1.

### 2.5. Image Quality Assessment

Acquired images were analyzed and assessed at the core CT reading center by experienced readers (level 3 readers, with more than 10 years of experience) in the central CT core lab at Lundquist Institute in Torrance, CA. All studies were read blindly by two expert physicians, with adjudication by consensus by the same two readers if there were disagreements related to stenosis, plaque severity or image quality. A 4-point Likert scale was employed to perform qualitative analyses of these scans, as conducted by Nakanishi et al. [3]: Poor quality—impaired image quality precluding appropriate evaluation of the coronaries due to significant misalignment artifacts, severe motion artifacts, severe image noise, or insufficient image contrast making it unable to evaluate the lumen. Fair quality—reduced image quality due to a lesser degree of image distortion caused by misalignment and motion artifacts, image noise, or insufficient image contrast. Good quality—presence of artifacts caused by motion, image noise, or decreased image contrast, but fully preserved ability to assess the lumen. Lastly, excellent quality—complete absence of artifacts with clear delineation of vessel lumen boundaries.

### 2.6. Quantitative Analysis

The signal-to-noise (SNR) and contrast-to-noise ratios (CNR) were computed to serve as objective indices of image quality. Mean density values of the contrasted aorta, measured by placing a circular region of interest in the center of the aorta, divided by the standard deviation of the contrasted aorta, defined the aorta SNR. Furthermore, the left ventricle CNR was computed by taking the difference between the mean density of the contrasted left ventricle and the mean density of the left ventricular wall (which served as a variable for the background signal), divided by the standard deviation of the contrast-filled left ventricle. Similarly, the CNR for the aorta was calculated by taking the difference between the mean density of the contrasted aorta and the mean density of the left ventricular wall, divided by the standard deviation of the contrasted aorta. The standard deviation of the respective regions of interest constituted image noise.

### 2.7. Statistical Analysis

Analyses were performed using statistical software (SAS version 9.1; SAS Institute, Cary, NC, USA). A statistically significant difference was defined as a *p*-value (two-tailed) of less than 0.05. Continuous variables were expressed as mean +/− SD. A two-sided *t* test and chi-squared tests were used to analyze the main outcomes. KVP and BMI were matched across cohorts.

## 3. Results

A total of 104 consecutive subjects with heart rates above 70 beats per minute were evaluated for this study. Out of the 104 patients, 52 underwent scans using the 4 cm scanner and 52 underwent scans using the 16 cm scanner. The mean heart rate was 75 ± 7 beats per minute in the 4 cm scanner and 75 ± 7 beats per minute in the 16 cm scanner (*p* = 0.426).

Table 2 includes the results of objective image quality analysis. Aorta SNR in the 16 cm scanner was 11.5 ± 4.2, and it was 8.7 ± 4 in the 4 cm scanner, marking a difference of 2.8 ± 4.1 (*p* = 0.0001) between the two modalities. Aorta CNR with the 16 cm scanner was 9 ± 3.7, and it was 6.3 ± 3.5 in the 4 cm scanner, with a difference of 2.7 ± 3.6 (*p* = 0.0002). Lastly, the left ventricle CNR difference between the 16 cm scanner and the 4 cm scanner was 2.7 ± 3 (*p* < 0.0001).

Table 3 demonstrates the subjective assessments of image quality for each respective scanner. The data show that more scans on the 16 cm scanner were evaluated as having an excellent quality in comparison with those performed on the 4 cm scanner (*p* < 0.0001). Overall, there were far fewer poor- or fair-quality images in the 16 cm arm of the study (5/52 = 9.6%) than in the 4 cm scanner arm (38/52 = 73%), with the majority of artifacts constituting stepwise and RCA motion artifacts.

## 4. Discussion

Historically, images acquired from subjects with heart rates above 70 bpm have resulted in significant motion and misalignment artifacts that affected CCTA diagnostic performance, which deemed them unfavorable and inadequate for analysis. However, recent advances in software and hardware capabilities have enabled the development of new-generation CCTA modalities that are capable of producing high-quality images, even in patients with high heart rates. As a result, we are now reconsidering the conventional belief that patients with high heart rates are unsuitable for CCTA analysis.

Our study demonstrates a superior image quality acquired on the 16 cm scanner in comparison to the 4 cm scanner in terms of both quantitative and qualitative measures. Improvements in motion artifacts are likely primary driving forces in this difference, given the Likert point scale’s emphasis on motion disturbances in the coronary vessels. It was noted that imaging from the 4 cm scanner showed significant coronary motion and stepwise artifacts relative to imaging conducted on the 16 cm scanner. Only 9.6% of the images in the 16 cm arm of the study constituted poor- or fair-quality images, as opposed to 73% of the images in the 4 cm scanner arm, as shown in Table 3 [4,5].

Moreover, SNR and CNR are important image-quality metrics in computed tomography. In our study, improvements in both SNR and CNR were observed on the latest generation of CT scanners compared to previous models, as demonstrated in Table 1. The increase in SNR and CNR was attributed to several factors, including the faster gantry rotation and improved temporal resolution of the newer scanner [6,7]. Additionally, iterative reconstruction and processing algorithms, such as GE Healthcare’s “SnapShot Freeze”, also contributed to the improved image quality [8,9]. However, the differences in CNR between the 16 cm and 4 cm scanners were mainly driven by the use of adaptive statistical iterative reconstruction (ASIR-V) on the newer scanner, which is a more advanced and efficient method compared to the traditional filtered back projection used on the 4 cm studies. Overall, the results suggest that the latest generation of CT scanners provides higher quality images with improved diagnostic accuracy and reduced radiation [10].

The utilization of the 16 cm scanner has led to a significantly increased frequency of good-quality images, with considerably fewer poor images, as demonstrated in Table 3 and as touched upon earlier. The improvement in scanner technology has been the primary driving force behind this. With the addition of more detectors, coverage has dramatically progressed, and we now have whole-heart coverage in a single rotation, which has achieved signal homogeneity from isophasic acquisition [4,5] and decreased artifacts to expand evaluation to multiple applications [11,12,13].

In the study by Ondrejkovic et al. [14], they evaluated patients with irregular heart rates and/or resting heart rates > 70 bpm with a 256 detector system (GE Healthcare, Milwaukee, WI, USA) similar to the system we utilized in our current study. They demonstrated improved image quality, and when specifically comparing those participants with a high heart rate (n = 104), they showed a good or excellent image quality in 90% of participants, exactly the same as in our current study (Table 4). They had a larger predominance of ‘good’ image quality as compared to ‘excellent’, while our study had a higher ‘excellent’ image quality. While this may just be a subjective difference by different observers, it is likely driven by the protocol. We extensively used oral beta blockers in our study, and they specifically state “No oral beta blockers were given.” Many studies have demonstrated that intravenous beta blockage in isolation in patients with elevated heart rates fails to result in ideal heart rate control, and this may be evident by comparing these two studies.

**Figure 1:**
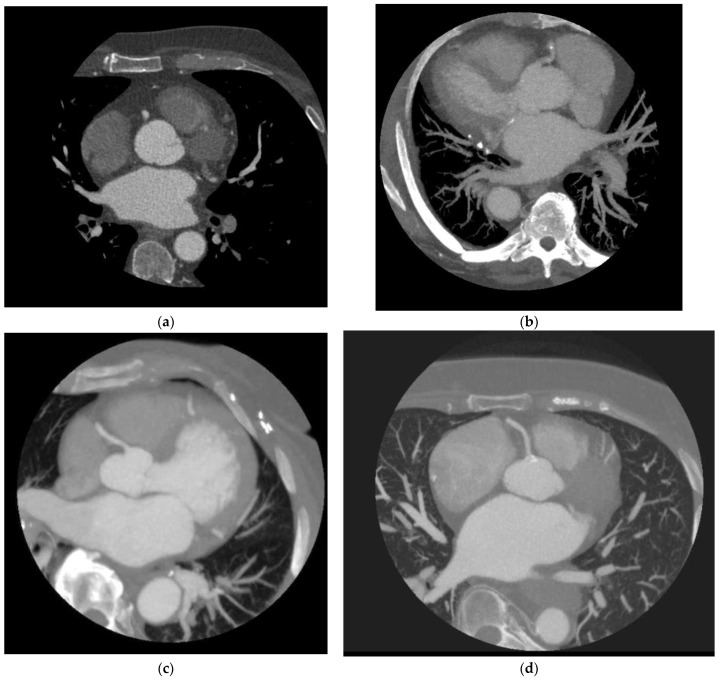
Acquisition of Patients with elevated heart rates with 16 cm (256) coverage. (**a**) A maximum intensity projection of the right coronary artery using a 16 cm scanner and heart rate of 82 bpm. (**b**) A maximum intensity projection of the right coronary artery using a 16 cm scanner and heart rate of 99 bpm. (**c**) A maximum intensity projection of the right coronary artery using a 16 cm scanner and heart rate of 75 bpm. (**d**) A maximum intensity projection of the right coronary artery using a 16 cm scanner and heart rate of 113 bpm.

**Figure 2:**
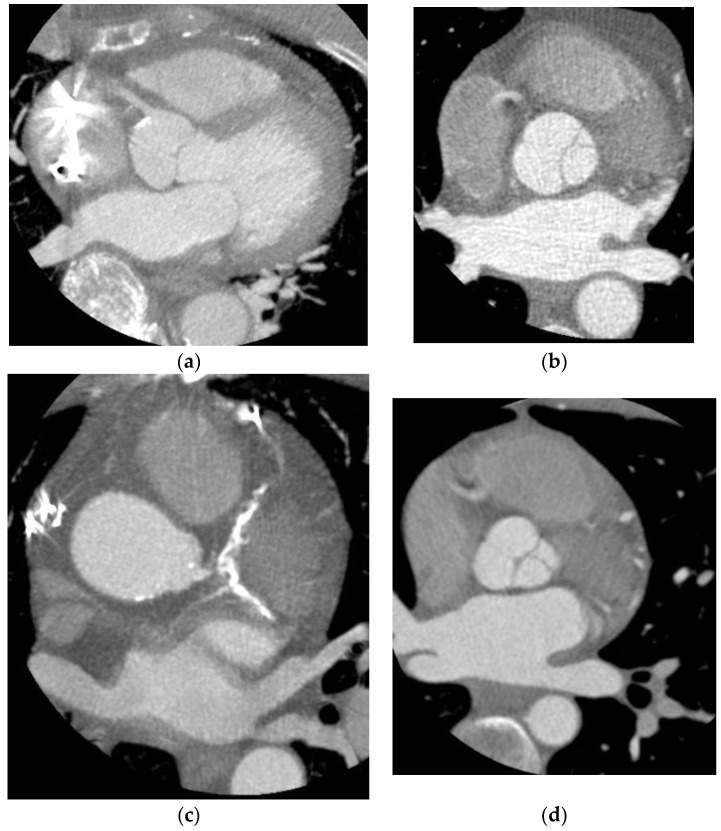
(**a**) A maximum intensity projection of the right coronary artery using a 4 cm scanner and heart rate of 70 bpm. (**b**) A maximum intensity projection of the right coronary artery using a 4 cm scanner and heart rate of 70 bpm. (**c**) A maximum intensity projection of the right and left coronary arteries using a 4 cm scanner and heart rate of 82 bpm. (**d**) A maximum intensity projection of the right and left coronary arteries using a 4 cm scanner and heart rate of 73 bpm.

The latest scanners have 256 detector rows, 16 cm cranial-caudal coverage, and a fast gantry rotation time of 280 ms, enabling acquisition of the entire heart within a single heartbeat using prospective triggering without table motion [7,8,9]. The wider coverage provided by the new scanners has allowed one to capture images during end-systolic phases of the cardiac cycle despite high heart rates, resulting in remarkable improvements in reducing motion artifacts (Figure 1 and Figure 2). The improved scanner technology has allowed for a better temporal resolution in the acquired images, which is vital for reducing motion artifacts. These advancements have paved the way for a better diagnostic performance in coronary CT scans by providing higher-quality images for analysis despite unfavorable heart rates.

There are several limitations to this study. The patients were not randomized, and the readers were not blinded; however, the cohorts in this study were matched based on age, BMI, KVP and gender. The design of the study was based on a prospective multi-center observational registry, which did not allow for patients to be scanned twice, given concerns of increased radiation exposure and IRB requirements. Additionally, the sample size on the 4 cm scanner is small, which is due to scans being aborted in light of unfavorable heart rates, whereas in the 16 cm scanner arm, a bigger portion of patients were scanned despite the higher heart rates. Future directions could include increasing the sample size, randomizing cohorts and blinding readers, as well as comparing image quality and motion in persons with unfavorable heart rates versus those in sinus rhythm on 16 cm scanners.

## 5. Conclusions

The 16 cm scanner has a superior image quality for fast heart rates (greater than 70 bpm) compared to the 4 cm scanner, with a significantly higher frequency of excellent and good studies and with better contrast-to-noise and signal-to-noise ratios, despite there being a lower radiation with the 16 cm scanner compared to the 4 cm scanner. Scans that were previously considered unfavorable on prior generations of CCTA modalities, given poor image acquisition due to inadequately controlled heart rates despite premedication with heart-lowering agents, are now deemed appropriate for analysis and may be included in future studies without the need to reimage and further expose patients to contrast.

## Figures and Tables

**Table 1:** Demographics and image-acquisition properties.

	Total	4 cm Scanner	16 cm Scanner	
Number of Subjects	104	52	52	
	Mean Std.	Mean Std.	Mean Std.	*p*
Age	60.6 ± 15.3	58.6 ± 16.8	62.6 ± 13.4	0.189
BMI	27.8 ± 5.1	27.9 ± 5.1	27.8 ± 5.2	0.912
Heart Rate	74.9 ± 7.2	75.1 ± 7.1	74.6 ± 7.4	0.426
Radiation dose	3.9 ± 4.5	5.6 ± 5.3	2.1 ± 2.6	<0.0001
kVp				
100	56	28	24	
120	48	28	24	
ASIR				
0	20	19	1	
30	21	21	0	
40	6	6	0	
50	7	5	2	
60	50	1	49	

**Table 2:** Quantitative analysis of image quality.

	Total	4 cm Scanner	16 cm Scanner	Difference	
Number of Subjects	104	52	52		
	Mean Std.	Mean Std.	Mean Std.	Mean Std.	*p*
Aorta Signal-to-noise	10.1 ± 4.3	8.7 ± 4.0	11.5 ± 4.2	(2.8) ± 4.1	0.0001
Aorta Contrast-to-noise	7.6 ± 3.8	6.3 ± 3.5	9.0 ± 3.7	(2.7) ± 3.6	0.0002
LV Contrast-to-noise	6.5 ± 3.3	5.2 ± 3.6	7.9 ± 2.1	(2.7) ± 3.0	<0.0001

**Table 3:** Qualitative assessment of image quality.

	Total	4 cm Scanner	16 cm Scanner
Number of Subjects	104	52	52
CTA Image Quality			
Excellent	41	4	37
Good	20	10	10
Fair	13	9	4
Poor	30	29	1

**Table 4:** Comparison of image quality.

		Ondrejkovic et al. [14]	Ondrejkovic et al. [14]
Number of Subjects	52	154	104 (Regular rhythm > 70 bpm)
	Mean Std.	Mean Std.	
Age	62.6 ± 16.8	62 ± 11	
Heart Rate	74.6 ± 7.1	80 ± 12	
Weight	87 ± 18	85 ± 16	
Radiation Dose	2.1 ± 2.6	3.0 ± 1.5	
Scanner	256 Row—16 cm	256 Row—16 cm	256 Row—16 cm
Beta Blocker	Oral and Intravenous	Intravenous Only	Intravenous Only
Male/Female	31/21	86/68	
Excellent	37 (71%)	22 (14%)	16 (15%)
Good	10 (19%)	101 (66%)	68 (65%)
Fair	4 (7.7%)	25 (16%)	17 (16%)
Poor	1 (1.9%)	6 (3.9%)	3 (2.9%)

## Data Availability

The data are available upon request from Dr. Budoff.

## Abbreviations

CCTA: coronary computed tomographic angiography; CONVERGE: comprehensive evaluation of high temporal and spatial resolution cardiac CT using a wide coverage system; bpm: beats per minute; SNR: signal-to-noise ratio; CNR: contrast-to-noise ratio; HR: heart rate.
